# Initial Alterations of Fish Community Structure and Diversity Following Fishing Cessation in Qilu Lake, China

**DOI:** 10.3390/ani14162289

**Published:** 2024-08-06

**Authors:** Tingbing Zhu, Feifei Hu, Jinling Gong, Kairun Liu, Zhibin Guo, Deguo Yang, Xuemei Li

**Affiliations:** 1 Key Laboratory of Freshwater Biodiversity Conservation, Ministry of Agriculture and Rural Affairs, Yangtze River Fisheries Research Institute, Chinese Academy of Fishery Sciences, Wuhan 430223, China; zhutb@yfi.ac.cn (T.Z.); 17612182178@163.com (F.H.); jlgong123@163.com (J.G.); singularity_gzb@163.com (Z.G.); yangdg@yfi.ac.cn (D.Y.); 2 Fishery Workstation of Tonghai County, Yuxi 652799, China; lkr002@126.com

**Keywords:** eutrophic lake, ecological fishery, fishing cessation, fish community structure, diversity

## Abstract

**Simple Summary:**

Ecological fishery management is one of the predominant approaches to lake ecological management in China; it involves the stocking of filter-feeding fish species to mitigate algal blooms. This strategy not only enhances water quality but also yields ecological benefits by fostering the growth of fish populations, thereby offering both ecological and economic advantages. Nonetheless, there is a scarcity of works in the literature addressing the effects on fish community dynamics and diversity in lakes subsequent to the cessation of ecological fishing practices. This study focuses on the initial changes observed in fish community composition and diversity following the cessation of an ecological fishery project in Qilu Lake, an eutrophic shallow lake situated in China. Ecological fishery projects aim to improve the water quality of eutrophication lakes by stocking filter-feeding fish species that feed on algae. The findings indicate a significant decline in the number of fish species, average individual size, and species diversity index following the cessation of fisheries-related activities (including fish stocking and fishing activities) due to the termination of the ecological fishery project in the lake. To preserve fish biodiversity and enhance water quality in Qilu Lake, it is recommended that policies be implemented to sustain the implementation of ecological fisheries projects, restore habitats for native fish species, and regulate the proliferation of small fish populations.

**Abstract:**

The Qilu Lake is an eutrophic shallow lake located in Yunnan Province, China. An ecological fishery project was initiated in the lake from 2011 to 2021 to introduce filter-feeding fish species that feed on algae, with the aim of improving water quality. In January 2022, when the ecological fishery project expired, all fisheries-related activities (including fish stocking and fishing activities) ceased in the lake. To comprehensively evaluate the initial alterations in fish community structure and diversity resulting from the fishing cessation in the Qilu Lake, the present study conducted field surveys within the one year before the fishing cessation (referred to as BFC) and the one year after the fishing cessation (referred to as AFC). A total of twenty-one fish species were collected, including four native species. Four species were recorded in the lake for the first time, including *Pelteobagrus fulvidraco*, *Pelteobagrus vachelli*, *Paramisgurnus dabryanus*, and *Hyporhamphus intermedius*. The number of fish species decreased from 21 to 13 following the fishing cessation. The fishes collected in both BFC and AFC are mainly omnivorous-feeding and of bottom-dwelling habits. The mean size of the fishes in the AFC sample shows a significant decrease compared to those in BFC. After the fishing cessation, the Shannon–Wiener diversity index and Margalef richness index of the fish slightly declined. The fish community structure of the Qilu Lake exhibits a high degree of similarity to adjacent lakes in central Yunnan. Our study demonstrates a significant shift in the fish community of the Qilu Lake following the fishing cessation, one which may adversely impact the stability of the lake ecosystem. To enhance fish species diversity in the Qilu Lake, it is recommended that policies be implemented to promote the ecological fishery project and improve habitat restoration for native fish species, while also regulating fish community structure.

## 1. Introduction

Eutrophication of lakes caused by human activities is a common issue in China. In the past 40 years, the area of eutrophic lakes in China has increased by a multiple of nearly 60 [1]. In response to this problem, Liu and Xie proposed the concept of “non-traditional bio-manipulation” (NTBM), which involves stocking filter-feeding fish species (such as silver carp *Hypophthalmichthys molitrix* and bighead carp *Aristichthys nobilis*) to directly graze on phytoplankton, aiming to control algal blooms in lakes [2,3]. As many successful cases have been achieved [2,4,5,6,7], the “non-traditional bio-manipulation” concept has become one of the concepts most frequently used for addressing eutrophication in lakes. Consequently, a large number of ecological fishery projects based on this concept have been implemented in the eutrophication-affected lakes of China.

The Qilu Lake, located in the central region of Yunnan Province, is a representative plateau shallow lake renowned for its abundant water and aquatic resources. Approximately 92.9% of the population in Tonghai County resides around the Qilu Lake, which supports irrigation across 96.7 km^2^ of farmland. The lake offers diverse functionalities including climate regulation, domestic water supply, industrial and agricultural water usage, water storage, flood control, navigation, tourism, and aquaculture [8]. Over time, the Qilu Lake has been transformed into a national wetland park due to its rich aquatic biological diversity. Historically, the lake was home to a variety of native fish species, such as *Cyprinus chilia*, *Cyprinus pellegrini*, *Cyprinus ilishaestomus*, *Cyprinus yunnanensis*, and *Anabarilius qiluensis*, which were predominant until 1958. Additionally, the lake harbors 39 species of aquatic plants, including the human-edible plant *Ottelia acuminata* [9]. Notably, benthic animals, particularly rare snails like *Margarya mansuyi*, have exhibited high biomass levels, with a recorded yield of 145 g/m^2^ in 1982 [10].

In recent years, human activities and the impacts of climate change have had significant detrimental effects on the aquatic ecosystem of Qilu Lake. Since 1958, water diversion initiatives have notably reduced the native fish population in the lake. Starting in 1964, various fish species, including the four main Chinese carps and goldfish (*Carassius auratus*), have been introduced annually. The agricultural sector surrounding the Qilu Lake has undergone substantial expansion, with some regions repurposed for farming, resulting in increased irrigation and notable agricultural pollution. Recent times have seen an increase in drought occurrences, leading to the drying-up of certain water sources. Consequently, the Qilu Lake has experienced a significant decrease in water supply, reduced surface area, lowered water levels, severe eutrophication, and a decline in water quality. The native fish population has rapidly declined, while invasive species have proliferated. Submerged vegetation is almost extinct, with only sporadic instances of emergent plants and a limited presence of pollution-tolerant submerged flora in the nearshore areas. The population of benthic organisms has also drastically declined, with scarce numbers of live mussels and snails observed, and only a small number of pollution-tolerant species such as oligochaetes and chironomid larvae. Guided by the “non-traditional bio-manipulation” concept, a ten-year (2011–2021) ecological fishery project initiative concentrating on stocking filter-feeding fishes was implemented with the purpose of controlling eutrophication and improving the water quality of the Qilu Lake. After the project concluded, all fishing activities in the lake were stopped and the economically stocked filter-feeding fish were removed, leading to the depletion of most major economically valuable fish species.

Research on the fish species diversity in the Qilu Lake has been lacking for over a decade. Previous studies mainly focused on identifying the fish species in the lake, leading to varying results. Li [11] identified nine native fish species in the Qilu Lake, including *C. chilia*, *C. pellegrini*, *C. ilishaestomus*, *C. yunnanensis*, *A. qiluensis*, *C. auratus*, *Misgurnus anguillicaudatus*, *Silurus asotus*, and *Monopterus albus*. Subsequently, He et al. [10], Gao et al. [12], Yang et al. [13], and Wang and Dou [14] documented 25, 10, 11, and 19 fish species in the same lake, respectively. The most recent study by Yuan et al. [15], conducted between 2007 and 2008, revealed a total of fourteen fish species in the lake, with only three native species. The extended research hiatus of nearly fifteen years has led to a lack of information regarding the community structure and diversity of fish species in the Qilu Lake. Consequently, the present study undertook field surveys in the Qilu Lake from 2021 to 2023 to evaluate the initial alterations in fish community structure and diversity, using samples taken before and after the fishing cessation. The purpose of the investigation is to establish a scientific basis for the conservation and sustainable use of fish species diversity in the Qilu Lake.

## 2. Materials and Methods

### 2.1. Study Area

**Figure 1:**
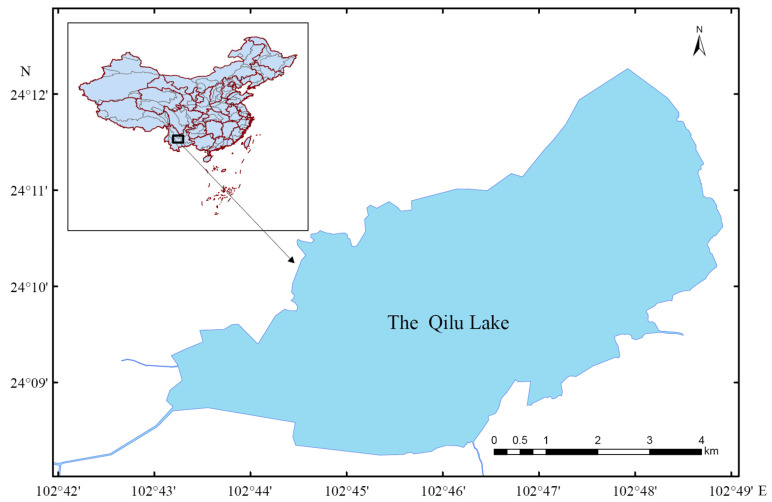
Geographical location of the Qilu Lake.

The Qilu Lake, located in southwestern China, falls within the geographical coordinates of 102°43′ to 102°49′ E and 24°08′ to 24°13′ N (Figure 1), and is a part of the Pearl River system. It is regarded as one of the central lakes in Yunnan, ranking as the sixth-largest among the nine principal plateau lakes in the province. At an elevation of 1796 m, the Qilu Lake spans a length of 10.4 km and reaches a maximum width of 4.4 km, covering an area of 38.86 km^2^. The lake boasts an average water depth of 4.03 m and a storage capacity of 1.486 billion m^3^ [14].

A ten-year (2011–2021) ecological fishery project was implemented in the Qilu Lake to control eutrophication and improve the water quality by stocking silver carp and bighead carp. Subsequently, intensive removal fishing for silver carp and bighead carp was conducted at the end of the project (December 2021 and January 2022) with the aim of reducing surplus nutrients such as nitrogen and phosphorus.

### 2.2. Sampling Method

A field investigation was conducted to collect data on the species composition, abundance, and biomass of fishes in the Qilu Lake. Sampling fishing was carried out within the one year before the fishing cessation (referred to as BFC) and the one year after the fishing cessation (referred to as AFC). The specific sampling times for BFC were January, March, June, July, September, and December 2021, while the specific sampling times of AFC were July and October 2022, and January 2023. The sampling sites are dynamic, yet they typically ensure uniform coverage across the entire lake. Fishing gear employed included compound gill nets (30 m in length, 2 m in height, mesh sizes (knot-to-knot) ranging from 1.0 cm to 11.0 cm) and trap nets (10 m in length, 25 cm in width, 30 cm in height, mesh size 7 mm). Gill nets were used across the entire lake, whereas trap nets were mainly deployed in the shallow waters of the nearshore zone. Each fishing survey involved leaving the fishing gear in the water for 4 to 24 h. Subsequently, the fish samples collected were initially identified by species and then measured for body length (to 0.1 cm), and body weight (to 0.1 g).

### 2.3. Data Analysis

#### 2.3.1. Alpha Diversity

The Shannon–Wiener diversity index (*H*′), Simpson dominance index (*D*), Margalef richness index (*R*), and Pielou evenness index (*J*′) were calculated independently for BFC and AFC. The calculation formulae for these indices are as follows:

(1) $$H^{\prime }=-\sum_{i=1}^{S}P_{i}lnP_{i}$$

(2) $$D=1-\sum_{i=1}^{S}{\left(P_{i}\right)}^{2}$$

(3) $$R=\frac{S-1}{lnN}$$

(4) $$J^{\prime }=\frac{H^{\prime }}{lnS}$$

where *H*′ is the Shannon–Wiener diversity index; *D* is the Simpson dominance index; *R* is the Margalef richness index; *J*′ is the Pielou evenness index; *S* is the total number of fish species in the community; and *P_i_* is the proportion of the total number of individuals that belong to species *i* (i.e., *Pi* = *n_i_*/*N*, where *n_i_* is the number of individuals of species *i* and *N* is the total number of all fish species).

The differences of the alpha diversity indices between BFC and AFC were tested using one-way ANOVA.

#### 2.3.2. Catch Per Unit of Effort (CPUE)

In order to further reveal the differences in the community structure between BFC and AFC, the Catch Per Unit Effort (CPUE) was calculated for each period independently. For gill nets, the CPUE is expressed as the weight of catches per square per hour (g/m^2^/h), while for trap nets, it is represented as the weight of catches per cubic meter per hour (g/m^3^/h).

#### 2.3.3. Community Similarity

The Jaccard similarity index was employed to evaluate the resemblances in fish community and species composition between the Qilu Lake and the adjacent lakes, as well as the main source-lakes of alien fish species in the middle and lower reaches of the Yangtze River, such as the Honghu Lake [16], the Dongting Lake [17], and the Taihu Lake [18]. The adjacent lakes include the Yangzonghai Lake [19], the Fuxian Lake [20], and the Yilong Lake [21].

The similarity index (*I*) was calculated as follows [22]:

*I* = *j*/(*a* + *b* − *j*) (5)

where *I* represents the similarity index between community *A* and *B*, *a* denotes the number of species in community *A*, *b* denotes the number of species in community *B*, and *j* represents the number of species shared between communities *A* and *B*. When *I* ranges from 0 to 0.25, the communities are extremely dissimilar; when *I* ranges from 0.25 to 0.5, they are moderately dissimilar; when *I* ranges from 0.5 to 0.75, they are moderately similar; when *I* ranges from 0.75 to 1.0, they are extremely similar.

## 3. Results

### 3.1. Fish Species Composition

A total of 21 species from 17 genera, six families, and four orders of fish were collected (Table 1). A total of four indigenous fish species were collected, including *C. chilia*, *C. pellegrini*, *C. auratus*, and *M. anguillicaudatus*, accounting for 19.0% of the total fish species collected. *Pelteobagrus fulvidraco*, *Pelteobagrus vachelli*, *Paramisgurnus dabryanus*, and *Hyporhamphus intermedius* were recorded in the lake for the first time (Table 1). Combined with the historical data, a total of 30 fish species have been found in the Qilu Lake, belonging to 23 genera, nine families, and seven orders, including 11 native species and 19 introduced species (Table 1). Species such as *C. pellegrini* are listed in the National Key Protected Wildlife List. In the China Biodiversity Red List, *A. qiluensis*, *C. yunnanensis*, *C. ilishaestomus*, and *C. pellegrini* are classified as critically endangered, while *C. chilia* and *Silurus grahami* are categorized as endangered.

The numbers of fish species recorded in BFC and AFC were 21 and 13, respectively. AFC had eight fewer species compared to BFC, with the absent species including *H. molitrix*, *A. nobilis*, *C. pellegrini*, *Ctenopharyngodon idella*, *Megalobrama amblycephala*, *S. asotus*, *Hypseleotris swinhonis*, and *P. vachelli* (Table 2).

The fishes’ feeding habits were 61.91% omnivorous, 19.05% carnivorous, 9.52% herbivorous, and 9.52% filter-feeding in BFC, compared to 76.92% omnivorous and 23.08% carnivorous in AFC (Table 2). The distribution of fish across water layers was 42.86% bottom layer, 28.57% upper-middle layer, and 28.57% lower-middle layer in BFC, while in AFC it was 46.15% bottom layer, 30.77% upper-middle layer, and 23.08% lower-middle layer (Table 2).

### 3.2. Fish Community Structure

In BFC, a collection of 2352 fish samples were taken, amounting to a total weight of 601.21 kg. In AFC, a total of 808 fish samples were collected, weighing 24.55 kg in total.

**Figure 2:**
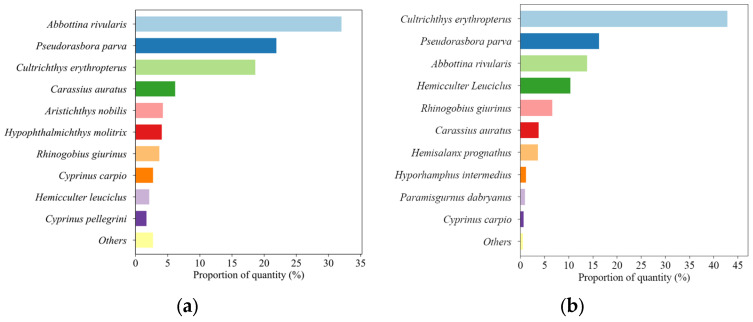
Changes in fishery-catch quantity proportions (**a**) before and (**b**) after the fishing cessation in the Qilu Lake.

For BFC, the quantity-leading species included *Abbottina rivularis*, *Pseudorasbora parva*, *Cultrichthys erythropterus*, *C. auratus*, *A. nobilis*, *H. molitrix*, *Rhinogobius giurinus*, *Cyprinus carpio*, *Hemicculter leuciclus*, *C. pellegrini* (Figure 2a). For AFC, the top species in quantity were *C. erythropterus*, *P. parva*, *A. rivularis*, *H. leuciclus*, *R. giurinus*, *C. auratus*, *Hemisalanx prognathus*, *H. intermedius*, *P. dabryanus*, and *C. carpio* (Figure 2b). Following the fishing cessation, there was an observed increase in the proportional abundance of fish species such as *C. erythropterus* and *H. leuciclus*. Conversely, there was a notable decrease in the proportions of certain species, including *A. rivularis* and *P. parva*.

**Figure 3:**
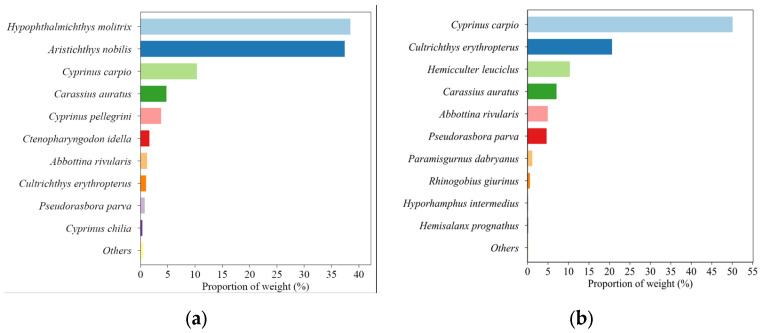
Changes in fishery-catch weight proportions (**a**) before and (**b**) after the fishing cessation in the Qilu Lake.

For BFC, the species most abundant in weight were *H. molitrix*, *A. nobilis*, *C. carpio*, *C. auratus*, *C. pellegrini*, *C. idella*, *A. rivularis*, *C. erythropterus*, *P. parva*, and *C. chilia* (Figure 3a). For AFC, the top species in weight were *C. carpio*, *C. erythropterus*, *H. leuciclus*, *C. auratus*, *A. rivularis*, *P. parva*, *P. dabryanus*, *R. giurinus*, and *H. intermedius* (Figure 3b). Following the fishing cessation, there was an observed increase in the weight proportions of *C. carpio*, *C. erythropterus*, and *H. leuciclus*.

In terms of gill nets, the CPUE in BFC and AFC were 3.8 g/m^2^/h and 2.8 g/m^2^/h, respectively. In terms of trap nets, the CPUE in BFC and AFC were 5.8 g/m^3^/h and 6.9 g/m^3^/h, respectively.

### 3.3. Fish Size Structure

The mean body length (Table 3) and body weight (Table 4) of fishes in the Qilu Lake significantly decreased after the fishing cessation, exhibiting a trend towards miniaturization. However, the majority of the residual small-sized fish species within AFC exhibited negligible discrepancies in mean body length and weight compared to those within BFC (Table 3 and Table 4).

### 3.4. Fish Diversity

**Figure 4:**
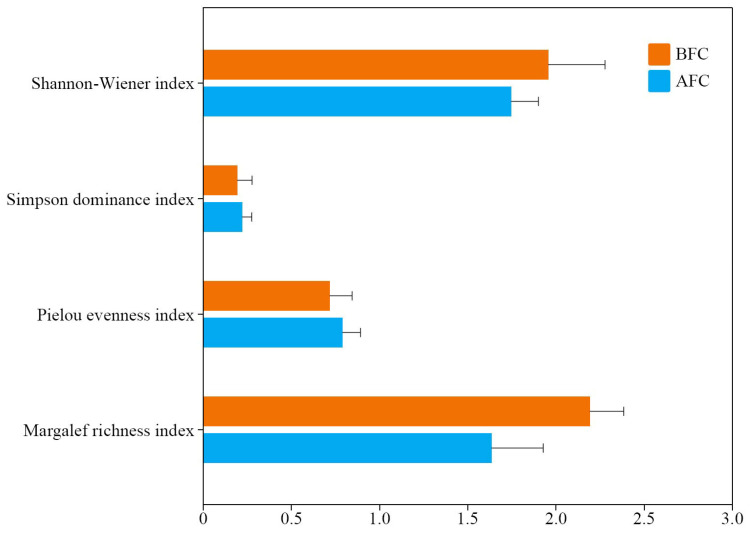
Alpha diversity of fish community in the Qilu Lake. BFC—before the fishing cessation; AFC—after the fishing cessation. Error bars represent the standard deviation.

There was no statistically significant difference in the alpha diversity indices between BFC and AFC (*p* > 0.05). Nevertheless, the mean values of the Shannon–Wiener diversity index and Margalef richness index were slightly higher in AFC compared to BFC (Figure 4).

### 3.5. Community Similarity Index

The fish community structure of the Qilu Lake was extremely dissimilar to that of the Dongting Lake, and moderately dissimilar to other studied lakes (Table 5). The highest similarity index was found in the Yangzonghai Lake (Table 5).

## 4. Discussion

Human activities in recent decades have severely disrupted the ecosystem of the Qilu Lake, leading to substantial changes in its fish community. Historical research and the current study indicate significant shifts in fish resources within the lake.

The fishery production activities in the Qilu Lake have experienced a gradual development process. Since 1964, fishery production in Qilu Lake has gradually increased, from 70 tons in 1980 [12] to 335 tons in 1985 [14], and an annual output of about 1000 tons was observed from 2005 to 2006, accounting for 70–80% of the total production [15]. According to statistical data from the Tonghai County Fisheries Work Station, the fishing productivity of the Qilu Lake from 2011 to 2020 was 1000 to 1500 tons. It can be seen that the fishery production activities in the Qilu Lake have lasted for a long time, but the purpose of developing fisheries has shifted from a focus on production to a focus on ecological management by using fishes to control algae.

The proliferation of alien fish species in the Qilu Lake has escalated due to ongoing fishing activities. Prior to 1958, the lake was primarily inhabited by native fishes such as *C. chilia*, *C. yunnanensis*, and *A. qiluensis*. However, the introduction of large-scale water extraction and lake reclamation in 1958 resulted in significant depletion of native fish populations. Subsequently, since 1964, various alien economically important fishes have been annually introduced into the lake, including *Mylopharyngodon piceus*, *C. idella*, *A. nobilis*, *H. molitrix*, *C. carpio*, *M. amblycephala*, and *C. auratus*. The number of alien fish species in the Qilu Lake has exhibited fluctuations over time, with 15 species reported in 1987 [10] and 12 species found in 2007–2008 [15]. In the present study, 17 alien fish species were collected. Despite variations in survey methodologies and levels of rigor across different studies, a discernible pattern emerges indicating a decline in native fish species within the Qilu Lake, with alien species gaining dominance. Notably, *C. pellegrini*, a native fish species in the lake, has been classified as a second-class nationally protected wild animal. Other native fish species such as *A. qiluensis*, *C. ilishaestomus*, and *C. yunnanensis* have not been recorded for an extended period, underscoring the urgent need for conservation research. Furthermore, the invasion of alien fishes has led to a convergence in fish fauna between the Qilu Lake and neighboring lakes such as the Fuxian Lake and the Yilong Lake, which exhibit high similarity in fish species composition. The stocking of introduced fish species in the Qilu Lake primarily comprises commonly cultured species from the middle and lower reaches of the Yangtze River, thereby contributing to the resemblance of its fish fauna to that of typical lakes in the same region.

The fish community structure of the Qilu Lake underwent notable changes following the fishing cessation. Before the fishing cessation, the dominant species in the lake were commercially valuable fishes such as *H. molitrix*, *A. nobilis*, *C. carpio*, and *C. auratus*. After the fishing cessation, the fish community shifted towards omnivorous and bottom-dwelling species including *C. carpio*, *C. erythropterus*, *H. leuciclus*, *C. auratus*, *A. rivularis*, and *P. parva*. The CPUE of gill nets in BFC was notably higher than that of AFC. Conversely, for trap nets, the CPUE in BFC was lower compared to that of AFC. This may reflect selective fishing which targeted large fishes inhabiting the middle and upper water layers, while also suggesting an increase in the population of smaller, bottom-dwelling fishes. Additionally, the average individual size of fishes in the Qilu Lake decreased significantly after the fishing cessation, particularly among commercial species, while small-sized non-economically-valuable fish species did not exhibit significant changes in size. Jia et al. [24] also found a decrease in fish size following fishing bans, suggesting that selective fishing practices (e.g., the removal of large fishes such as *H. molitrix* and *A. nobilis*) may contribute to the observed changes in fish community structure after fishing cessation.

The biodiversity of the fish in Qilu Lake experienced significant changes following the fishing cessation. Although no statistically significant difference was found, there was a notable decrease in the fishes’ Shannon–Wiener diversity index and Margalef richness index, indicating a trend towards simplified fish community. The observed alterations in fish biodiversity can likely be attributed to the intensive removal of economically valuable fish species such as *H. molitrix*, *A. nobilis*, *C. idella*, and *M. amblycephala* during the end of the ecological fishery project, which resulted in a significant decrease of economic fish resources in the lake in 2022, when fishing activities were stopped. Species like *C. pellegrini*, *S. asotus*, *P. vachelli*, and *H. swinhonis* appeared to have disappeared after the fishing cessation, possibly due to their historically limited resources and the challenges associated with sample collection.

The ecological fishery project conducted in Qilu Lake is essentially a form of NTBM, distinct from the traditional biomanipulation (TBM) practices observed in areas like Europe and North America. The biological theory basis for biomanipulation is the top-down trophic cascade effect, which is widely applied in the management of eutrophic water bodies worldwide. Usually, TBM involves the addition or removal of benthivorous, zooplanktivorous, and piscivorous fishes to manage populations of grazing zooplankton [25], while NTBM involves stocking filter-feeding fish species to directly graze on phytoplankton [2,3]. Numerous studies have validated the efficacy of both TBM [26] and NTBM [2,4,5,6,7]. However, a limited number of studies have been conducted on the alterations in fish communities following biological manipulation or food web management. Rask et al. [27] found that the responses in the fish community structure and fish abundance to long-term mass removal of planktivorous fishes from eutrophicated Lake Tuusulanjärvi were slight and mostly masked by changes in the reproduction and growth of fishes and annual variability in environmental conditions. For a variety of factors, the present study observed the alterations in the fish communities in Qilu Lake only for one year before and after the end of biological manipulation. Although long-term monitoring is required to draw definitive conclusions, our study may still be helpful in inspiring research on the response of fish communities after biological manipulation.

The Qilu Lake plays important roles in climate regulation, landscape preservation, and other aspects in central Yunnan. Despite its importance, the lake faces various ecological challenges that require attention, including water pollution, eutrophication, depletion of native fish resources, proliferation of invasive species, and loss of aquatic plants. In the context of conserving and utilizing fish resources, several pertinent issues warrant consideration.

Firstly, it is imperative to optimize the development of ecological fisheries that utilize fishing as a means to control the overabundance of algae in Qilu Lake. The ecological fishery project primarily aims to diminish the algae population in eutrophic water bodies by introducing filter-feeding fishes, particularly *H. molitrix* and *A. nobilis*, thereby enhancing water quality. Concurrently, the regular harvesting and sale of the stocked fishes on land can not only eliminate surplus nutrients like nitrogen and phosphorus but also generate economic gains. The inadequate management of non-point-source pollution from agriculture has led to a persistent issue of eutrophication in Qilu Lake, which has been characterized by a notably high concentration of algae [28]. The total nitrogen concentrations in Qilu Lake were measured at 2.68 mg/L in BFC and 2.43 mg/L in AFC. Additionally, the total phosphorus concentrations in Qilu Lake were recorded as 0.26 mg/L in BFC and 0.35 mg/L in AFC, indicating that both nutrients are present at relatively elevated levels. Therefore, further biomanipulation is still required. It is suggested that, following a scientific evaluation of the lake’s carrying capacity, further refinement of the fish-stocking combination approach can be implemented to achieve more efficient management of Qilu Lake’s water quality.

Secondly, efforts should be made to restore the habitats of native fishes and strengthen their natural reproductive capabilities. The majority of native fish in Qilu Lake lay adhesive eggs, necessitating the use of aquatic plants for egg laying during natural reproduction. However, extensive water pollution over the years has resulted in the widespread decline of aquatic plants in the lake, hindering the fulfillment of the natural reproduction requirements of native fishes. Therefore, in addition to the strategy of “fishing to control algae”, it is imperative to implement rigorous pollution control measures and restore fish habitats. The restoration methods may include gradually replenishing aquatic plants, constructing artificial fish nests, and other interventions aimed at bolstering the natural reproduction of native fishes and facilitating the gradual recovery of their populations.

Thirdly, the management of fish community in the Qilu Lake is also necessary. After the fishing cessation, the fish community in the Qilu Lake is now primarily composed of small-sized and bottom-dwelling fishes, most of which are alien species. However, there exists a deficiency in regulatory measures targeting small-sized fishes in the Qilu Lake. The frequent disturbance of lake sediment by a large number of bottom-dwelling fishes for feeding or excretion purposes can notably elevate nitrogen and phosphorus levels in the water, resulting in issues like decreased water transparency. Previous studies have shown that the introduction of benthic fish species can triple the total phosphorus content compared to a fishless system and increase algae biomass significantly [29]. Furthermore, small-sized fishes like *P. parva* and *R. giurinus* consume substantial quantities of native fish eggs, exacerbating the decline of native fish resources. Generally, two strategies can be employed to manage small-sized fish populations: direct fishing and the introduction of predatory fishes. Given the challenges and expenses associated with directly fishing small-sized fish due to their size and elusive behavior, the more prevalent approach involves introducing predatory fishes to regulate the surplus of small-sized fish resources. This method not only incurs lower labor costs but also allows for the conversion of small-sized fish resources into valuable predatory fish products through the food chain, thereby generating economic advantages. It is advisable to introduce predatory fish species such as mandarin fish *Siniperca chuatsi*, which have limited natural reproduction in the lake, to control the small-sized fish population, subsequent to a comprehensive evaluation of small-sized fish resource levels in Qilu Lake.

## 5. Conclusions

In summary, the present study found a significant decline in the number of fish species, average individual size, and species diversity index subsequent to the cessation of ecological fishing activities in the Qilu Lake. To preserve fish biodiversity and enhance water quality in Qilu Lake, it is recommended that policies be enacted to sustain the implementation of ecological fisheries projects, restore habitats for native fish species, and regulate the proliferation of small-sized fish populations.

## Figures and Tables

**Table 1:** Composition of fish species in the Qilu Lake.

Order	Family	Scientific Name	Distribution Record ^1^
Cypriniformes	Cyprinidae	*Hypophthalmichthys molitrix*	△▲
		*Aristichthys nobilis*	△▲
		*Ctenopharyngodon idella*	△▲
		*Mylopharyngodon piceus*	△
		*Anabarilius qiluensis*	○
		*Cyprinus carpio*	△▲
		*Cyprinus chilia*	○●
		*Cyprinus pellegrini*	○●
		*Cyprinus yunnanensis*	○
		*Cyprinus ilishaestomus*	○
		*Carassius auratus*	○●
		*Parabramis pekinensis*	△
		*Megalobrama amblycephala*	△▲
		*Cultrichthys erythropterus*	△▲
		*Abbottina rivularis*	△▲
		*Hemicculter leuciclus*	△▲
		*Rhinogobius giurinus*	△▲
		*Hypseleotris swinhonis*	△▲
		*Pseudorasbora parva*	△▲
	Cobitidae	*Misgurnus anguillicaudatus*	○●
		*Paramisgurnus dabryanus*	▲
Beloniformes	Adrianichthyidae	*Oryzias latipes*	○
Siluriformes	Bagridae	*Pelteobagrus fulvidraco*	▲
		*Pelteobagrus vachelli*	▲
	Siluridae	*Silurus asotus*	△▲
		*Silurus grahami*	○
Synbranchiformes	Synbranchidae	*Monopterus albus*	○
Perciformes	Channidae	*Channa argus*	○
Salmoniformes	Salangidae	*Hemisalanx prognathus*	△▲
Beloniformes	Hemiramphidae	*Hyporhamphus intermedius*	▲

^1^ ○ represents native species with historical records, ● represents native species found in this study, △ represents alien fish species with historical records, and ▲ represents alien fish species found in this study. Historical distribution records are referenced from [10,11,12,13,15,23].

**Table 2:** Ecological types of fish in the Qilu Lake.

Species	Feeding Habits	Habitat Water Layers	Sampled in BFC	Sampled in AFC
*Hypophthalmichthys molitrix*	Filter-feeding	Middle upper	+	
*Aristichthys nobilis*	Filter-feeding	Middle upper	+	
*Ctenopharyngodon idella*	Herbivorous	Middle and lower	+	
*Cyprinus carpio*	Omnivorous	Middle and lower	+	+
*Cyprinus chilia*	Omnivorous	Middle and lower	+	+
*Cyprinus pellegrini*	Omnivorous	Middle and lower	+	
*Carassius auratus*	Omnivorous	Bottom	+	+
*Megalobrama amblycephala*	Herbivorous	Middle and lower	+	
*Cultrichthys erythropterus*	Carnivorous	Middle upper	+	+
*Abbottina rivularis*	Omnivorous	Bottom	+	+
*Hemicculter leuciclus*	Omnivorous	Middle upper	+	+
*Rhinogobius giurinus*	Carnivorous	Bottom	+	+
*Hypseleotris swinhonis*	Omnivorous	Bottom	+	
*Pseudorasbora parva*	Omnivorous	Middle upper	+	+
*Misgurnus anguillicaudatus*	Omnivorous	Bottom	+	+
*Paramisgurnus dabryanus*	Omnivorous	Bottom	+	+
*Pelteobagrus vachelli*	Omnivorous	Bottom	+	+
*Pelteobagrus fulvidraco*	Omnivorous	Bottom	+	
*Silurus asotus*	Carnivorous	Bottom	+	
*Hemisalanx prognathus*	Carnivorous	Middle and lower	+	+
*Hyporhamphus intermedius*	Omnivorous	Middle upper	+	+

+ represents samples of the respective fish species have been collected.

**Table 3:** Comparison of body lengths of fish in the Qilu Lake before and after the fishing cessation.

Species	Body Length in BFC			Body Length in AFC		
	Range (cm)	Mean (cm)	*n*	Range (cm)	Mean (cm)	*n*
*Cultrichthys erythropterus*	5.4–27.8	10.0	438	7.3–22.4	10.8	346
*Pseudorasbora parva*	4.0–10.8	7.7	515	2.5–10	7.6	128
*Abbottina rivularis*	3.9–11.1	7.8	753	4.0–10.6	8.3	111
*Hemicculter leuciclus*	8.8–17.2	11.7	50	4.6–18.7	12.7	83
*Rhinogobius giurinus*	3.0–7.1	5.1	87	2.5–7	5.2	52
*Carassius auratus*	3.6–38	14.9	145	3.7–18.4	9.6	30
*Hemisalanx prognathus*	7.3	7.3	1	6.4–8.7	7.4	29
*Hyporhamphus intermedius*	12.5–14.3	13.7	4	9.7–15	12.8	9
*Paramisgurnus dabryanus*	9.2–18.1	14.2	16	11.1–22.4	14.7	7
*Cyprinus carpio*	4.2–51	28.5	64	29.8–49	39.1	5
*Cyprinus chilia*	10.0–33	21.6	4	8.3–8.4	8.4	2
*Pelteobagrus fulvidraco*	7.3–8.2	7.8	2	4.2	4.2	1
*Misgurnus anguillicaudatus*	10.0–13.2	11.4	3	8.6	8.6	1
*Hypophthalmichthys molitrix*	34.5–71	48.2	96			
*Aristichthys nobilis*	32.0–71	46.7	100			
*Cyprinus pellegrini*	4.5–45	13.8	40			
*Hypseleotris swinhonis*	2.8–4.9	3.9	29			
*Ctenopharyngodon idella*	60.0–70	65.0	2			
*Megalobrama amblycephala*	40.0	40.0	1			
*Pelteobagrus vachelli*	17.8	17.8	1			
*Silurus asotus*	14.4	14.4	1			
Total	2.8–71.0	12.7	2352	2.5–49.0	9.84	804

**Table 4:** Comparison of body weights of fish in the Qilu Lake before and after the fishing cessation.

Species	Body Weight in BFC			Body Weight in AFC		
	Range (cm)	Mean (cm)	*n*	Range (cm)	Mean (cm)	*n*
*Cultrichthys erythropterus*	2.8–305	13.6	438	3.8–160.1	14.6	346
*Pseudorasbora parva*	0.5–20.6	8.5	515	0.2–17.8	8.7	131
*Abbottina rivularis*	0.9–24.8	9.6	753	0.7–22.9	11.0	111
*Hemicculter leuciclus*	7.3–73.5	19.3	50	0.8–94.9	30.6	83
*Rhinogobius giurinus*	0.4–5.1	2.3	87	0.2–6.7	2.7	53
*Carassius auratus*	1–1400	195.5	145	1.3–198	58.0	30
*Hemisalanx prognathus*	1.7	1.7	1	0.9–1.9	1.4	29
*Hyporhamphus intermedius*	4.2–5.9	5.1	4	1.5–8.5	5.0	9
*Paramisgurnus dabryanus*	6.6–59	28.9	16	13.4–140.6	40.7	7
*Cyprinus carpio*	1.6–3700	967.7	64	649.9–4470	2459.3	5
*Cyprinus chilia*	23.2–900	461.7	4	12.9–13.7	13.3	2
*Pelteobagrus fulvidraco*	7.3–10.2	8.8	2	1.2	1.2	1
*Misgurnus anguillicaudatus*	4.7–23.6	12.6	3	5.3	5.3	1
*Hypophthalmichthys molitrix*	836.5–7800	2404.5	96			
*Aristichthys nobilis*	600–7000	2248.6	100			
*Cyprinus pellegrini*	1.8–3400	560.7	40			
*Hypseleotris swinhonis*	0.2–1.7	0.8	29			
*Ctenopharyngodon idella*	4000–5800	4900.0	2			
*Megalobrama amblycephala*	1700.0	1700.0	1			
*Pelteobagrus vachelli*	86.8	86.8	1			
*Silurus asotus*	36.3	36.3	1			
Total	0.2–7880.0	255.6	2352	0.2–4470.0	30.4	808

**Table 5:** Comparison of fish community similarities between the Qilu Lake and other lakes.

Lake	Number of Fish Species	Quantity of Species Shared with the Qilu Lake	Community Similarity Index
Fuxian Lake [20]	33	14	0.350
Yangzonghai Lake [19]	19	12	0.429
Yilong Lake [21]	10	8	0.348
Honghu Lake [16]	42	14	0.286
Dongting Lake [17]	69	15	0.200
Taihu Lake [18]	68	18	0.254

## Data Availability

Data was contained within the article and will be made available upon request.
